# Endoscopic Ultrasound-Guided Tissue Acquisition Using Fork-Tip Needle Improves Histological Yield, Reduces Needle Passes, Without On-Site Cytopathological Evaluation

**DOI:** 10.1089/pancan.2018.0018

**Published:** 2018-10-31

**Authors:** Zhigang Song, Charles N. Trujillo, Helen Song, Jane E. Tongson-Ignacio, Michael Y. Chan

**Affiliations:** ^1^ Division of Gastroenterology, Department of Internal Medicine, Kaiser Permanente Fontana Medical Center, Fontana, California.; ^2^ Department of Surgery, Kaiser Fontana Medical Center, Fontana, California.; ^3^ Kaiser Permanente Fontana Medical Center, Fontana, California.; ^4^ Department of Cytology, Southern California Kaiser Permanente Regional Reference Laboratories, North Hollywood, California.

**Keywords:** endoscopic ultrasound-guided fine-needle aspiration, tissue acquisition, fine needle aspiration needle, fork-tip fine needle biopsy needle, rapid on-site cytopathology evaluation

## Abstract

**Background and Aim:** Endoscopic ultrasound (EUS)-guided fine needle biopsy (FNB) and fine needle aspiration (FNA) are established methods in tissue acquisition. A new fork-tip FNB needle has been used to obtain core tissue samples. We compared the performance of the FNB using fork-tip needles with that of the FNA using conventional needles in patients who had solid neoplastic lesions within and around the upper gastrointestinal (GI) tract.

**Methods:** In this retrospective single-center study, patients who underwent EUS examinations for solid neoplastic lesions between October 2013 and February 2017 were included. The procedures were performed in the absence of an on-site cytologist. The main objectives were to compare the diagnostic yield and average number of passes of FNB using fork-tip needles versus those of FNA using conventional needles.

**Results:** EUS/FNA and EUS/FNB were performed on 181 solid neoplastic lesions primarily in the pancreas and GI tract walls. There was no significant difference in patient's age, gender, tumor location, or tumor size. The mean number of needle passes was significantly lower in the fork-tip needle group than in the conventional needle group (3.8 vs. 5.9; *p* < 0.0001). There was a trend toward higher sensitivity (89.9% vs. 81%) using the fork-tip needles than when using the conventional needles (*p* = 0.119). No significant difference in rates of adverse events between two groups was found.

**Conclusions:** Our study demonstrates that, compared with FNA using conventional needles, FNB using fork-tip needles required significantly fewer needle passes while achieving a relatively higher diagnostic yield due to its superior capacity in tissue acquisition from solid neoplastic lesions in and around GI tract walls without on-site cytological assessment.

## Introduction

Tissue acquisition using endoscopic ultrasound (EUS) is an established method for sampling solid lesions within and around the upper gastrointestinal (GI) tract. EUS-guided fine needle aspiration (FNA) has been described for sampling the pancreas, bile duct, liver, adrenal gland, lymph nodes, and subepithelial lesions within the wall of the GI tract. The average sensitivity and specificity of EUS-FNA for solid pancreatic neoplasms is 85% and 96%, respectively.^1–13^ Optimal EUS-guided tissue acquisition depends on many factors, including endosonographer experience, needle type, needle gauge (G), number of passes, fanning technique, use of syringe suction, lesion size, lesion location, and presence of rapid onsite cytopathology evaluation (ROSE).^1,14^

Although much progress has been made to improve EUS-FNA tissue sampling, limitations of the technique include inadequate preservation of tissue architecture and insufficient material to perform immunohistochemistry, which are vital for the diagnosis of lymphomas, neuroendocrine tumors, mesenchymal tumors, and autoimmune pancreaticobiliary diseases. In addition, ROSE has been shown to decrease the number of needle passes,^15^ but many community medical centers may not have expert cytopathologists available.^1^

Core needles for EUS-guided fine needle biopsy (FNB) could theoretically bypass the shortcomings of traditional EUS-FNA. Initial core needles did demonstrate significantly improved diagnostic yield compared with its FNA counterparts.^16,17^ More recently, the development of a fork-tip needle, the SharkCore needle (Medtronic, Inc., Minneapolis, MN), has been demonstrated to require the same or fewer number of passes to achieve a diagnosis while providing significantly more histological yield.^18–20^ Fewer needle passes using EUS-FNB could lead to decreased procedure time, adverse events, and cost, while providing the benefit of increased histological yield.

Although the initial studies involving the SharkCore needle are quite promising, it remains unclear whether its performance can be replicated in the community setting without ROSE. The aim of our retrospective study is to compare the outcomes of standard needles versus those of fork-tip (SharkCore) needles in solid neoplastic lesions.

## Methods

This is a retrospective single-center study of consecutive outpatients and inpatients who underwent EUS examinations from October 2013 to February 2017. All procedures were performed in the endoscopy unit of Kaiser Permanente Fontana Medical Center, a tertiary care hospital that is part of a large integrated health organization. The study was approved by the local institutional review board. Informed consent was obtained for all patients before the procedures.

Two endosonographers (Z.S. and M.Y.C.) performed the EUS examinations using a linear-array echoendoscope (GF-UCT/P-180 series; Olympus America, Melville, NY) with patients under conscious sedation or general anesthesia. EUS procedures were reviewed in the electronic health record. Patients who had cystic lesions or procedures without FNA/FNB were excluded.

Both FNA using conventional needles (Expect Slimline; Boston Scientific, Marlborough, MA, and EchoTip Ultra; Cook Medical, Bloomington, IN) and FNB using the fork-tip needles (SharkCore; Medtronic, Boston, MA) were performed with standard techniques. Suction with a syringe and capillary technique and fanning method were used. The average number of to-and-fro strokes on each pass was 15 to 18 times. Smears were made on the slide and preserved in 100% alcohol. The remainder of tissue samples was preserved in formalin for cell blocks. The target lesion was visualized with EUS. Color Doppler was used to determine an avascular path. Because our cytologists were located in a different facility, ROSE was not available for our procedures.

When using FNA with conventional needles, we adopted seven passes, if feasible, as suggested by previous studies.^21–24^ When using FNB with the fork-tip needles, we aimed to obtain similar quantity of tissue samples that was the FNB end-point. At conclusion of the procedures, slides and cell block were sent to the Southern California Permanente Medical Group Regional Reference Laboratory in Los Angeles where they were reviewed by experienced cytopathologists. Presence of definitive neoplastic cells was reported as a positive result. Others, such as suspicious for malignancy, atypical cells, or scant cellularity, were considered negative results. Patients with negative results on the first EUS examination were scheduled for repeat EUS/FNA or FNB or subsequent surgical biopsies until definitive diagnosis was established.

### Statistical analysis

All analyses were conducted using SAS version 9.4 (SAS Institute, Inc., Cary, NC). Descriptive analyses were performed on patient demographic and clinical characteristics. Statistical tests were conducted using *t*-tests and chi-square tests where appropriate. All reported *p* values are two sided.

## Results

### Patients and tumor characteristics

A total of 170 consecutive patients were included during the study period. There were 41 patients in conventional needle group and 129 patients in fork-tip needle group. There were no significant differences between the two groups with respect to age, gender, and tumor size (*p* > 0.05) (Table 1).

**Table 1.** **Clinical Characteristics of Patients**

	Fork-tip FNB (*n* = 139)	Conventional FNA (*n* = 42)	*p*
Age, mean (SD)	64.7 (11.9)	61.2 (12.6)	0.107
Gender, female (%)	52.5	45.2	0.408
Tumor size, mean (SD)	28.0 (14.4)	28.2 (18.0)	0.948
Tumor type (%)			0.608
Pancreas	77.7	78.6	
Subepithelial	20.1	16.7	
Other	2.2	4.8	

FNA, fine needle aspiration; FNB, fine needle biopsy; SD, standard deviation.

**FIG. 1.**
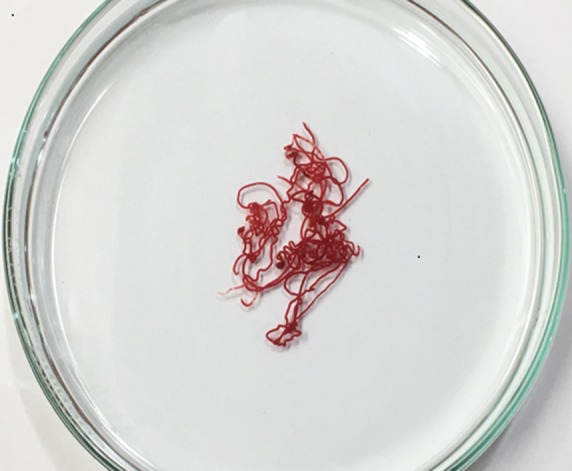
Cores of tissues acquired with one pass using 22G fork-tip needle; placed in 10 cm petri dish.

**FIG. 2.**
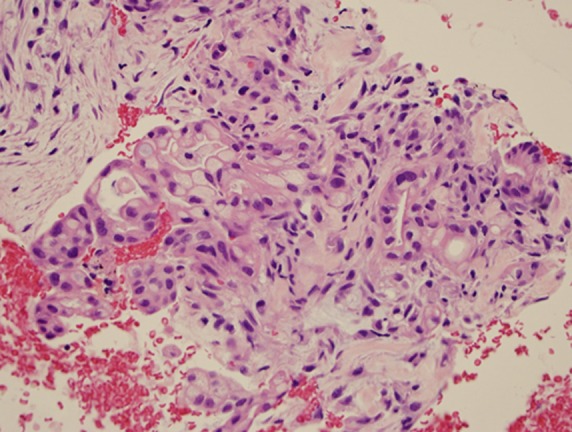
Adenocarcinoma of pancreas; hematoxylin and eosin (40** ×** ).

**FIG. 3.**
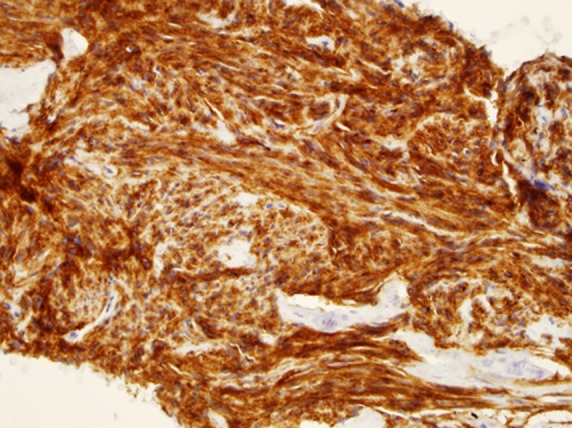
CD117 staining; gastrointestinal stromal tumor of stomach (40** ×** ).

**FIG. 4.**
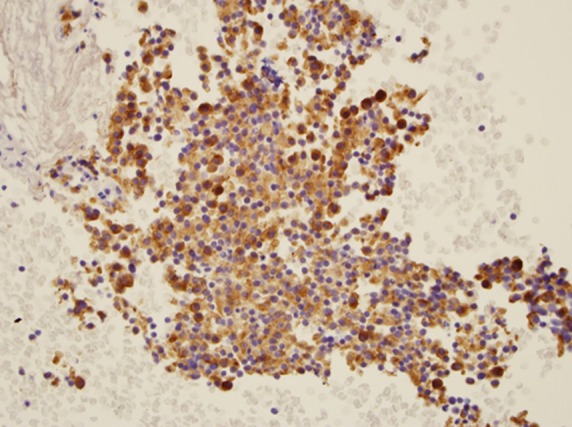
Synaptophysin staining; neuroendocrine tumor of pancreas (40 × ).

In the conventional needle group, one patient had two simultaneous lesions who underwent FNA, resulting in a total of 42 lesions. In the fork-tip needle group, 10 patients had two simultaneous lesions who underwent FNB, resulting in a total of 139 lesions. Based on their locations, they were classified as pancreatic lesions, subepithelial lesions in GI tract, and others. In the conventional needle group, there were 32 pancreatic lesions (76.2%), 8 subepithelial lesions in GI tract (19%), and two other lesions (metastatic lesions in the left lobe of liver, 4.8%). In fork-tip needle group, there were 111 pancreatic lesions (80.4%), 25 subepithelial lesions in GI tract (18.1%), and two other lesions (one metastatic carcinoma in lymph node and one adenoma in distal common bile duct, 1.5%) (Figs. 1–4).

There was no significant difference between two groups in terms of their tumor location (*p* > 0.05). In conventional needle group, the pancreatic lesions included 20 ductal adenocarcinomas (47.6%), 11 neuroendocrine tumors (26.2%), and 1 other lesion (large B cell lymphoma, 2.4%). In fork-tip needle group, there were 87 ductal adenocarcinomas (63%), 17 neuroendocrine tumors (12%), and 7 other lesions (1 large B cell lymphoma, 3 metastatic carcinomas, and 3 solid pseudopapillary neoplasms, 5%).

### Diagnostic yield of EUS/FNA and EUS/FNB

Forty-two solid lesions underwent EUS/FNA using either 25G or 22G FNA needles. Because ROSE was not available, we observed tissue cores acquired from each pass as an indicator to estimate the quantity of tissues (Fig. 1). The overall sensitivity of FNA using conventional needles was 81%. Owing to this suboptimal sensitivity, we subsequently switched to FNB using the fork-tip FNB needles. One hundred thirty-nine consecutive solid lesions underwent EUS/FNB using either 25G or 22G fork-tip needles. The overall sensitivity of using the fork-tip needles was 89.9% (*p* = 0.119). The average number of passes using conventional needles was 5.9 ± 2.09 compared with 3.8 ± 1.28 using fork-tip needles (*p* < 0.0001) (Table 2).

**Table 2.** **Number of Passes and Diagnostic Yield**

	Fork-tip FNB	Conventional FNA	*p*
Overall	*n* = 139	*n* = 42	
No. of passes, mean (SD)	3.8 (1.3)	5.9 (2.1)	<0.0001
Diagnostic yield	89.9%	81.0%	0.119
25G	*n* = 47	*n* = 34	
No. of passes, mean (SD)	3.4 (1.2)	5.5 (1.7)	<0.0001
Diagnostic yield	87.2%	82.4%	0.542

Thirty-four solid lesions underwent EUS/FNA using a 25G FNA needle. The sensitivity of using 25G FNA needle was 82.4% compared with 87.2% using 25G fork-tip FNB needle (*p* = 0.54). The average number of passes using 25G FNA needle was 5.5 ± 1.73 compared with 3.4 ± 1.19 using 25G fork-tip FNB needle (*p* < 0.0001) (Table 2).

Minor bleeding was the only observed adverse event in both conventional needle group and fork-tip needle group. In the FNA group using conventional needles, there was one patient who had self-limited minor bleeding. In the FNB group using fork-tip needles, four patients were found to have minor bleeding that did not require any intervention. There was no significant difference between these two groups (*p* = 1).

## Discussion

The significance of this study is threefold. First, we demonstrated a significant reduction of the number of needle passes for tissue acquisition on solid neoplastic lesion by using new fork-tip needles compared with conventional needles. The average number of saved passes was 2.1 when using fork-tip needles (3.8 passes) versus regular needles (5.9 passes).

These findings were consistent with some recent studies. In a meta-analysis by Khan et al.^25^ to compare fork-tip needles with conventional needles, 15 studies and a total of 1024 patients were included. FNB using different core needles was found to have fewer passes than FNA using regular needles. In another recent study by El Chafic et al.,^26^ conventional needles were compared with fork-tip needles on sampling of GI stromal tumors. Fewer needle passes were required using fork-tip needles than using conventional needles.

This reduction of the number of needle passes has an important impact on our practice of EUS/FNA and EUS/FNB. With fewer number of passes, it clearly shortened the procedural time and lessened cost of the examination. In addition, fewer needle passes could potentially decrease the risks of the procedures, such as bleeding, infection, pancreatitis, and perforation. More importantly, the reduction of number of needle passes using fork-tip needles did not compromise on diagnostic yield.

In our study, fork-tip needles achieved relatively higher sensitivity, 89.9% versus 81%, compared with conventional needles. Given the small sample size of the FNA group using conventional needles, it was not surprising to find no statistically significant difference between the diagnostic yields of these two groups. The explanation for the small sample size of the conventional needle group was its suboptimal performance. The tedious process of obtaining high number of passes and suboptimal diagnostic yield using conventional needles prompted us to seek alternative needles. Once realizing the improved performance of the fork-tip needles, we continued to use them on solid neoplastic lesions.

In fact, a better sensitivity and accuracy using the fork-tip needles were shown in a study by Nayar et al.^27^ when comparing them with a core needle from another manufacturer. In this study, the fork-tip needles were found to provide substantially higher sensitivity and accuracy in sampling solid pancreatic masses.

Second, all EUS/FNA and EUS/FNB procedures included in this study were carried out without an on-site cytologist. The value of on-site cytology assessment has much been discussed. Some studies showed the on-site cytologist can reduce the number of passes.^1,14,15^ However, most of EUS/FNA and FNA/FNB procedures across the country are performed without the on-site cytologist. Therefore, the optimal number of total passes in this setting is largely unknown.

A recent study by Lee et al.^28^ showed that seven passes without an on-site cytologist was not inferior to the presence of an on-site cytologist. It was noted that the procedural time in obtaining seven passes can be longer than when fewer passes were made. In our study, by using fork-tip needles, we demonstrated a steady trend of reducing the number of passes while still maintaining a high diagnostic yield in the absence of an on-site cytologist.

It is worth mentioning that a large variety of tumor locations and types were sampled. These lesions were not only located in the pancreas but also in and around the walls of the GI tract. From the aspects of cytopathology, as illustrated in our study, pancreatic ductal adenocarcinoma neuroendocrine tumors of the pancreas and smooth muscle GI tract tumors (e.g., GI stromal tumors and leiomyomas) were the most commonly diagnosed neoplasms, whereas rare and often difficult to diagnose lesions, such as B cell lymphoma and metastatic lesions in the pancreas, were also included. These findings are considered to be valuable to endosonographers who search for a reliable and efficient needle in tissue acquisition during EUS examination, especially without on-site cytological assessment.

It was noted that significant amount of visible microcores of the tissue samples was obtained using fork-tip needles. There has also been a trend to obtain more preserved tissue samples on cytological assessment by certified cytologists. These findings should become more relevant when attempting to make diagnoses of difficult lesions, such as metastatic tumors or lymphomas, which require well-preserved tissue architecture and sufficient quantity for immunohistochemical staining. As a result, during all our EUS/FNB procedures, we have been using the presence of visible cores to serve an additional indication that adequate tissue has been obtained. Further study is planned with the aim to quantitate the visible cores of tissues to determine the optimal number of passes using fork-tip needles.

Finally, there used to be concerns about the safety of EUS core needles. Many recent studies have shown that rates of adverse events remained acceptably low using fork-tip needles. Our study showed that self-limited minor bleeding was the only observed complication with 25G and 22G fork-tip needles, with no significant difference compared with conventional needles.

In our practice, pancreatitis and abdominal pain were seen in small number of patients who underwent EUS/FNB for other indications, such as cystic lesions in pancreas. No incidence of pancreatitis or other adverse events was observed in these patients likely because the targets were solid lesions. Our adverse event rates were comparable with other recent studies.^18,19,20,27^ Therefore, this study suggested that it is safe to use fork-tip needles on solid neoplastic lesions in pancreas and GI tract.

There are limitations to our study in large part due to its retrospective design. It was a serial design, FNA needle group followed by FNB needle group. It was not a randomized study. We tried to minimize selection bias by the inclusion of consecutive patients. In addition, this study was conducted at a single center, which may limit its generalizability. However, it should be noted that two certified endosonographers performed the procedures independently. Moreover, it reflects the realistic aspects of EUS/FNA and EUS/FNB, in the setting where an on-site cytologist is not available.

One more limitation could be the nonblind design of cytologists to the method of tissue acquisition. It was noted that all FNA or FNB specimens were randomly assigned to three different certified cytologists. All cytologists were not notified the switch from conventional needles to fork-tip needles before the conclusion of this study. Another potential limitation was lack of final surgical pathology to determine the true accuracy of EUS results. To address this issue, we used strict criteria, presence of definitive neoplastic cells, for the positive results. All others, including suspicious for malignancy and atypical cells, were considered negative results. Lastly the sample size of conventional needle group was relatively small.

Therefore, a randomized prospective study in multiple centers with a larger sample size is needed to further confirm the performance of fork-tip EUS needles.

## Conclusion

Our study demonstrated that fewer needle passes were required to achieve adequate specimens using the new fork-tip FNB needles than the conventional FNA needles when sampling solid neoplastic lesions of the pancreas, GI tract, and extraluminal areas. Moreover, there appeared to be a trend toward higher diagnostic yield using fork-tip needles than using conventional needles.

## Abbreviations Used

EUS: endoscopic ultrasound
FNA: fine needle aspiration
FNB: fine needle biopsy
GI: gastrointestinal
